# Analysis of children with familial short stature: who should be indicated for genetic testing?

**DOI:** 10.1530/EC-23-0238

**Published:** 2023-09-19

**Authors:** Lukas Plachy, Lenka Petruzelkova, Petra Dusatkova, Klara Maratova, Dana Zemkova, Lenka Elblova, Vit Neuman, Stanislava Kolouskova, Barbora Obermannova, Marta Snajderova, Zdenek Sumnik, Jan Lebl, Stepanka Pruhova

**Affiliations:** 1 Department of Pediatrics, 2nd Faculty of Medicine, Charles University in Prague and University Hospital Motol, Prague, Czech Republic

**Keywords:** familial short stature, next-generation sequencing, growth plate disorders, GH treatment, predictors of monogenic short stature

## Abstract

Familial short stature (FSS) describes vertically transmitted growth disorders. Traditionally, polygenic inheritance is presumed, but monogenic inheritance seems to occur more frequently than expected. Clinical predictors of monogenic FSS have not been elucidated. The aim of the study was to identify the monogenic etiology and its clinical predictors in FSS children. Of 747 patients treated with growth hormone (GH) in our center, 95 with FSS met the inclusion criteria (pretreatment height ≤−2 SD in child and his/her shorter parent); secondary short stature and Turner/Prader–Willi syndrome were excluded criteria. Genetic etiology was known in 11/95 children before the study, remaining 84 were examined by next-generation sequencing. The results were evaluated by American College of Medical Genetics and Genomics (ACMG) guidelines. Nonparametric tests evaluated differences between monogenic and non-monogenic FSS, an ROC curve estimated quantitative cutoffs for the predictors. Monogenic FSS was confirmed in 36/95 (38%) children. Of these, 29 (81%) carried a causative genetic variant affecting the growth plate, 4 (11%) a variant affecting GH–insulin-like growth factor 1 (IGF1) axis and 3 (8%) a variant in miscellaneous genes. Lower shorter parent’s height (*P* = 0.015) and less delayed bone age (BA) before GH treatment (*P* = 0.026) predicted monogenic FSS. In children with BA delayed less than 0.4 years and with shorter parent’s heights ≤−2.4 SD, monogenic FSS was revealed in 13/16 (81%) cases. To conclude, in FSS children treated with GH, a monogenic etiology is frequent, and gene variants affecting the growth plate are the most common. Shorter parent’s height and BA are clinical predictors of monogenic FSS.

## Introduction

Familial short stature (FSS) is a term commonly used in clinical practice to describe the vertical transmission of a growth disorder. A short child is classified as having FSS if at least one of his/her parents is also short (height −2 SD or less in both the child and his or her shorter parent) (1). The etiology of FSS is heterogeneous (1, 2). Traditionally, polygenic inheritance is presumed in FSS (3). Genome-wide association studies (GWAS) have identified hundreds of genetic variants affecting body height. Each of the variants alone has only a small effect on human growth but may cause short stature if combined (4). However, recent studies have challenged this dogma by proving that monogenic growth disorders are substantially more frequent than previously expected (3).

According to the paradigm presented by Baron, the growth plate plays a key role in short-stature pathogenesis (5). Single-gene variants that influence the growth plate are found not only in children with clear clinical signs of bone dysplasia but also in many children with nonsyndromic short stature (1, 6). A typical example is a deficiency in SHOX protein that was found in 2–15% of individuals originally classified as having idiopathic short stature (ISS) (7). Moreover, heterozygous mutations in the *NPR2* gene were revealed in 2–6% of ISS children (8, 9, 10, 11), and various collagenopathies have recently been presented as a frequent cause of nonsyndromic familial short stature (12). Other genes essential for normal growth plate function include *FGFR3*, *ACAN*, *COMP*, *MATN3* and multiple genes encoding components of the RAS-MAPK signaling pathway (e.g. *NF1*, *PTPN11*, *SOS1*) (5, 13). Mutations in all these genes are transmitted mainly in an autosomal dominant (AD) matter and may thus cause monogenic FSS.

Apart from growth plate disorders, heterozygous mutations in genes affecting the growth hormone (GH)–insulin-like growth factor (IGF) axis are also known to cause familial short stature (6). Growth hormone deficiency (GHD) type II (*GH1* gene) is one example. Another possibility is rare AD inheritance of mutations in genes affecting pituitary morphogenesis and differentiation (e.g. *POU1F1*, *GLI2*, *LHX4*, *PITX2* and *HESX1*) that could cause FSS and multiple pituitary hormone deficiency (14, 15). Insensitivity to GH (IGF deficiency and IGF resistance) may also be inherited in an AD manner (e.g., *IGF1R*, *GHR* and *STAT5B* genes) (2, 16).

Despite the substantial advances in understanding the genetic background of short stature, most short children with short parents do not know the real cause of their growth disorder and are classified using only the descriptive diagnosis of FSS (1). The results of our pivotal study performed on children with severe FSS showed that monogenic etiology may be frequent among families with FSS and that growth plate disorders may be the leading cause of monogenic FSS despite no apparent bone dysplasia signs (1). However, the questions of whether detailed genetic testing should be used in routine clinical practice and who should eventually be indicated for genetic evaluation remain unanswered.

Finding a genetic cause of a growth disorder is important both to understand the etiopathogenesis of short stature in the family and to screen for possible comorbidities frequently associated with the specific genetic finding (2). According to expert opinion, several clinical features, such as severe short stature, low birth parameters or clinical evidence of bone dysplasia, are thought to be associated with a higher likelihood of a monogenic etiology of short stature (2). However, no study validating these clinical predictors has been published to date. Therefore, we expanded the number of children with FSS who were treated with GH that were examined in our pivotal study (1) to, first, confirm the results of the pivotal study concerning the proportion of monogenic etiology of FSS and its etiopathogenesis and, second, determine the clinical predictors for monogenic FSS.

## Materials and methods

### Patients

#### Inclusion criteria

The database of children treated with GH in our center currently includes 747 individuals. After the exclusion of patients with Turner syndrome, Prader-Willi syndrome, and those with secondary causes of their short stature (e.g. chronic renal insufficiency and secondary GH deficiency due to intracranial tumor and/or irradiation), 528 children remained for further evaluation. Within this group, 125 children had FSS defined as a life-minimum height ≤−2 SD in both the patient and his/her shorter parent. In 95 children with FSS, their legal guardians consented to genetic testing, and those children were enrolled in the study. All study participants or their legal guardians signed written informed consent prior to genetic testing. The study was approved by the institutional Ethics Committees of the 2nd Faculty of Medicine, Charles University in Prague, Czech Republic.

#### Clinical evaluation prior to the study

The heights of all children were obtained during anthropometric measurements that also focused on body proportionality (sitting height to total height ratio). Information about their birth parameters was obtained from medical records. The heights of all the parents were measured to the nearest 1 mm, and the heights of more distant relatives were obtained from the parents. All the data were standardized according to recent normative values (17, 18, 19). GHD and being born small for gestational age (SGA) were evaluated according to current guidelines. In all short children with auxological data suggestive of GHD and/or IGF1 levels below −2 SD using reference ranges standardized for age and sex, GH provocation tests were performed. Children with peak GH concentration below 10 ug/L in two different provocation tests were classified as GHD (20). Sex-steroid priming was used in all children of 7 years of age or older. Children with birth weight and/or birth length below −2 SD using the reference ranges standardized for sex and gestational week who showed no evidence of catch-up growth at the age of 4 years (height ≤−2.5 SD and growth velocity before treatment <0 SD) were treated with GH in SGA indication (21). Bone age (BA) was evaluated using the Tanner–Whitehouse method (22).

The median age of 95 children with FSS at inclusion in the study was 12 years (IQR 9–15 years), and their life-minimum height was −3.0 SD (−3.5 to −2.7 SD) and the height of their shorter parent was −2.7 SD (−2.9 to −2.2 SD). The children had been treated with GH for 5 years (3–7 years), with an average dose during the first year of treatment of 33 µg/kg/day (31−35 µg/kg/day). Within this group, 64/95 (67%) children were classified as having GHD. Their maximum GH level after stimulation was 6.4 µg/L (median; IQR 4.8−7.8 µg/L). Forty-seven children had mild GHD with stimulated GH concentrations of 5−10 µg/L, and the remaining 17 children had stimulated GH concentrations <5 µg/L. Fifty-one children (54%) within the study cohort were born SGA (23 children were born SGA for both length and weight, 25 for length only, and 3 for weight only). Their median birth weight and birth length were −2.0 (IQR −2.5 to −1.6) and −2.6 SD (IQR −3.1 to −2.3), respectively. Twenty children (20%) were classified as having combined GHD and SGA.

### Genetic testing

#### Genetic testing in routine clinical practice prior to the study

All patients underwent basic genetic testing prior to the study, as described previously (1). In all girls, Turner syndrome and SHOX haploinsufficiency were examined by fluorescence *in situ* hybridization. In all boys with confirmed disproportionate short stature, SHOX deficiency, including point mutations, was examined using Sanger sequencing and multiplex ligation-dependent probe amplification. In children with clinical suspicion of a specific genetic disorder, targeted genetic testing was performed. In 11 children, genetic diagnosis of FSS was elucidated prior to the study (genes *SHOX* (6), *ACAN* (2), *PTPN11* (2), and *NF1*). Children with no genetic cause of their short stature elucidated prior to the study were subsequently examined using next-generation sequencing (NGS) methods.

#### Next-generation sequencing methods

Genomic DNA was extracted from peripheral blood in all patients included in the study. DNA from the first 26 patients with severe FSS (life-minimum height <−2.5 SD both in the patient and his/her shorter parent) was analyzed using whole-exome sequencing, and DNA from the remaining 58 patients was analyzed using a custom-targeted NGS panel of 398 genes known to be associated or potentially associated with growth (Supplementary Table 1, see section on supplementary materials given at the end of this article). The genetic analysis was described in detail in our previous studies (1, 8).

### Evaluation of the genetic results

All variants with potential clinical importance obtained from NGS were confirmed using Sanger sequencing as described previously (23) and were subsequently evaluated using American College of Medical Genetics and Genomics (ACMG) standards and guidelines (24). For variant evaluation were also used the ACMG criteria implemented into the VarSome software that scores each ACMG rule as very strong, strong, moderate or supporting based on ACMG recommendations and data from the annotation (25). In some cases, the strength of the rules was modified according to the extended investigation of various databases and clinical evaluation of the patient. For the assessment of the segregation of genetic variants with short stature within their families, DNA and height information about additional relatives was obtained. The guidelines formulated by Jarvik *et al.* were followed (26) in the application of co-segregation in the pathogenicity classification. Finally, all variants were classified as pathogenic (P), likely pathogenic (LP), benign (B), likely benign (LB) or as variants of uncertain significance (VUS).

### Searching for the clinical predictors of monogenic etiology of FSS

The difference in selected clinical parameters (see Table 1) between children with a proven monogenic etiology of FSS (pathogenic or likely pathogenic genetic variant elucidated in genes known to cause short stature) and those without monogenic etiology of FSS were evaluated using a nonparametric test (Kruskal–Wallis test). We used a receiver operating characteristic (ROC) curve to estimate the cutoff of quantitative predictors characterizing the children with monogenic etiology of FSS (the highest Youden index). All tests were performed in MedCalc version 19 (MedCalc Software 2020). Statistical significance was defined as *P* < 0.05.

**Table 1:** Differences between children with monogenic FSS compared to those with no monogenic FSS etiology elucidated.

	Monogenic FSS	No monogenic etiology elucidated	*P*-value
Height before GH treatment (SD)	−3.1 (−3.6 to −2.7)	−3.0 (−3.5 to −2.7)	0.52
Height 1 year of GH treatment (SD)	−2.6 (−2.9 to −2.2)	−2.5 (−2.9 to −2.1)	0.71
Height 3 years of GH treatment (SD)	−2.1 (−2.6 to −1.5)	−1.8 (−2.3 to −1.5)	0.33
Sitting height-to-height ratio (SD)	0.8 (0.3–2.0)	0.9 (0.3–1.5)	0.58
Birth weight (SD)	−1.6 (−2.2 to −1.1)	−1.6 (−2.2 to −0.7)	0.61
Birth length (SD)	−2.0 (−3.0 to −1.4)	−2.1 (−2.7 to −1.4)	0.88
Shorter parent’s height (SD)	−2.8 (−3.2 to −2.4)	−2.5 (−2.9 to −2.2)	0.015
Growth velocity prior to GH (cm/year)	4.9 (4.0–5.5)	5.2 (4.1–5.9)	0.49
Growth velocity in the first year of GH treatment (cm/year)	8.8 (7.8–9.3)	8.6 (7.4–9.3)	0.33
Age at first endocrinological examination (years)	5 (3–7)	5 (4–7)	0.85
Age at GH treatment initiation (years)	7 (5–9)	6 (5–9)	0.49
Average GH dose in the first year of treatment (ug/kg/day)	33 (32–36)	33 (31–35)	0.08
Average GH dose in the first year of treatment, SHOX-D excluded (ug/kg/day)	33 (32–35)	33 (31–35)	0.39
BA before GH treatment (difference with CA, years)	−0.3 (−1.6 to +0.1)	−1.2 (−1.9 to −0.5)	0.026
IGF1 (SD)	−1.4 (−1.8 to −1.0)	−1.5 (−2.0 to −1.1)	0.63
Stimulated GH concentration (ug/L)	6.2 (4.6–8.6)	7.2 (5.3–8.8)	0.23

Values are expressed as medians and interquartile ranges. For statistical evaluation, ANOVA Kruskal–Wallis test was used.

BA, bone age; CA , calendar age; FSS, familial short stature; GH, growth hormone; SD, standard deviation; SHOX-D, SHOX deficiency.

## Results

**Figure 1:**
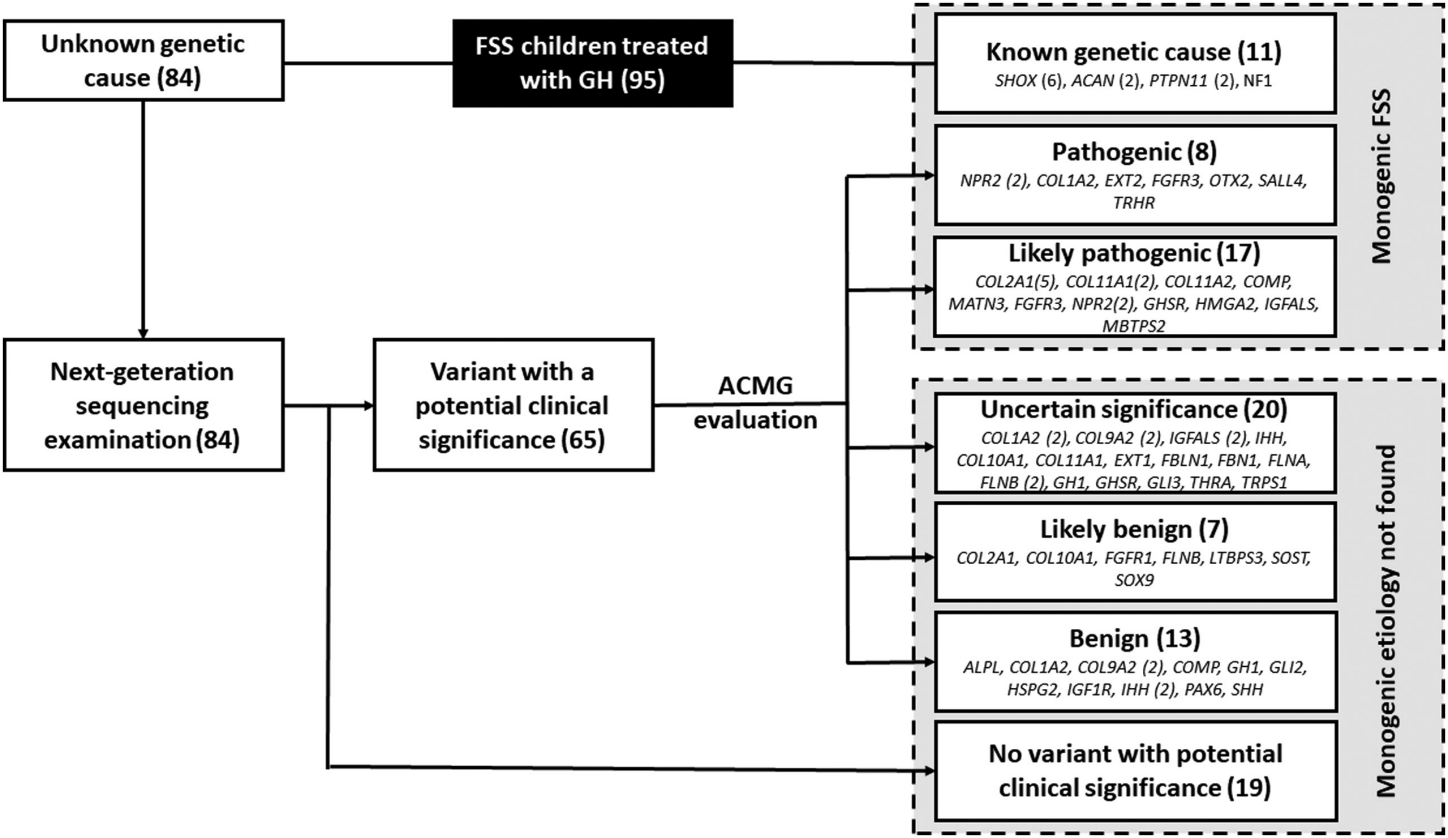
A workflow of the genetic testing process. ACMG, American College of Medical Genetics and Genomics standards and guidelines; FSS, familial short stature; GH, growth hormone.

Altogether, in 76/95 (80%) children with FSS treated with GH, we found at least one genetic variant of potential clinical importance in genes with a known impact on growth. Finally, monogenic etiology (pathogenic or likely pathogenic genetic variants discovered) was described in 36/95 (38%) children with FSS. Of these, 29 children (81%) carried causative genetic variants affecting the growth plate (*SHOX* (6), *COL2A1* (5), *COL11A1* (2), *NPR2* (4), *ACAN* (2), *FGFR3* (2), *PTPN11* (2), *COL11A2*, *COL1A2*, *COMP*, *MATN3*, *EXT2* and *NF1* genes), and 4 children (11%) carried variants affecting the GH–IGF1 axis (*GHSR*, *HMGA2*, *IGFALS* and *OTX2* genes). The remaining three children carried variants in miscellaneous genes (*TRHR*, *SALL4* and *MBTPS2* genes). In all children, heterozygous variants were inherited from their shorter parent. One exception was a pathogenic variant in the *SALL4* gene in patient 35 (short stature associated with radial ray defect), who inherited the variant not from his short mother (height −2.4 SD) but from his father with normal height (−1 SD) and radial ray defects. Thus, we presume that the *SALL4* pathogenic variant is causative for radial ray defects but likely not for short stature in the family. A diagram showing the examination process in detail is shown in Fig. 1. The specific causative genetic variants and the clinical characteristics of individual children are summarized in Supplementary Table 2, and the variants without proven causality (13 benign variants, 7 likely benign variants and 20 variants of uncertain significance) are summarized in Supplementary Table 3.

Compared to children with no elucidated monogenic etiology, children with monogenic FSS had significantly shorter affected parents and less delayed BA (Table 1). Using the ROC curve, the optimal cutoff values for monogenic prediction were shorter parent height of less than −2.4 SD (Youden index 0.29), and BA delay of less than 0.4 years (Youden index 0.34). Children who met the above criteria were found to have monogenic FSS in 81% (13/16 children). Importantly, other evaluated clinical parameters, including birth parameters, body disproportionality, severity of growth disorder or response to GH treatment, did not differ significantly in children with monogenic FSS compared to those with no monogenic etiology of FSS elucidated (Table 1).

## Discussion

Our study showed that monogenic etiology may be hidden among children with FSS and that the prevalence of such cases is not negligible. The growth disorder in 38% of FSS children from our study cohort was explained by a monogenic diagnosis. This is in line with the results of our pivotal study performed on 33 children with severe FSS. In this study, monogenic etiology was found in 52% of cases, and half of the children with monogenic FSS had a growth plate disorder, despite no apparent clinical signs of bone dysplasia (1). After we increased the number of patients in the study, the representation of children with monogenic FSS remained high; in addition, the prevalence of growth plate disorders was even more predominant (81%, 29/36 cases with monogenic FSS).

Although the prevalence of monogenic causes of short stature seems to be higher than originally expected, the results of genetic studies using NGS methods searching for monogenic growth disorders vary greatly, as monogenic short stature was elucidated in 9–54% cases (1, 27, 28, 29, 30, 31, 32). These great discrepancies suggest an urgency for clinical predictors of monogenic short stature that would help to select suitable patients for genetic testing. Importantly, clinical criteria have been created for some specific growth disorders, e.g., the Netchine-Harbison clinical scoring system for Silver–Russel syndrome (33) and the scoring system for Noonan syndrome (34). However, no evidence-based general predictors of monogenic short stature have been elucidated to date. In our study, we found that BA and parental height might be important factors. Surprisingly, the severity of short stature, body disproportionality or birth parameters suggested by Dauber *et al.* (2) did not prove to be suitable predictors of monogenic etiology in our study cohort.

Finding a monogenic cause of a growth disorder is important from both a clinical and scientific point of view. After obtaining the genetic diagnosis, the clinician and the family will finally obtain the answer as to why the child is not growing normally, and they may focus on possible hidden comorbidities associated with the genetic finding (e.g. congenital heart defect in Noonan syndrome or early osteoarthritis in *ACAN* gene mutations) (2, 13, 35). However, the treatment implications of genetic diagnosis are currently limited. Among children with proven monogenic etiology of their short stature, only those with SHOX deficiency and Noonan syndrome are known to benefit from GH treatment, and the genetic diagnosis automatically indicates them for therapy (36, 37, 38). On the other hand, the effect of GH treatment in children with achondroplasia is rather mild, and starting therapy is therefore controversial (39). In children with other monogenic causes of short stature, information about the efficacy of GH therapy is sparse, and genetic diagnosis currently does not bring any benefits related to therapy. Moreover, in some European countries, genetic diagnosis automatically excludes the child from the possibility of being treated for SGA/GHD indications, although no data prove worse efficacy of GH therapy in monogenic disorders. In our study, the effect of GH treatment did not differ significantly between children with proven monogenic etiology of their FSS and those with no monogenic cause elucidated (see Table 1). Thus, the authors suggest that no child should be excluded from the possibility of GH treatment because of SGA/GHD indications until the gene-specific response to GH treatment is elucidated.

Clarifying the genetic disorder-specific reactions to GH treatment that would lead to further refinement of indications for GH therapy poses one of the important current challenges of pediatric endocrinology. To achieve this aim, it will be necessary to accumulate large cohorts of children with specific monogenic growth disorders (8, 40). The results of our study might contribute substantially by identifying a relatively large group of children with great potential for having monogenic short stature that could serve as a source of patients for future studies. Moreover, understanding the spectrum of genetic mutations leading to short stature could aid in finding new therapies directly targeting the underlying pathophysiology of growth disorders in children not responding sufficiently to GH treatment (40).

For the abovementioned reasons, we consider genetic examination an important part of evaluating children with short stature. First, we should exclude Turner syndrome in all girls (optimally using the FISH method to capture possible mosaicism) and provide a targeted examination in case of clinical features leading to a suspicion of a specific genetic diagnosis (most frequently SHOX-deficiency, Silver–Russel syndrome or Noonan syndrome) (2, 33, 34). In case these examinations do not clarify the etiology of short stature, NGS methods should be considered. Based on our results, we recommend NGS examination in all children with FSS fulfilling the criteria for GH treatment if the socioeconomic situation allows it. Apart from FSS, other candidate groups for the NGS examination might include, i.e., children born SGA with persistent short stature (41), clinical signs of bone dysplasia (32) or congenital hypopituitarism (42). Specific clinical features predicting monogenic short stature including the ones we described must be confirmed by further studies.

We acknowledge that our study had several limitations. First, no functional studies have been performed. However, according to current guidelines, other methods can be used to prove the pathogenicity of genetic variants (24). In our cohort of patients with FSS, the most important of these was the segregation of the variants in patients with short stature within their families. The supportive methods used to evaluate the genetic variants included determining their frequency in population databases or various in silico studies. Secondly, protein noncoding variants (except for disruptions in the exon–intron boundaries) were not captured by the NGS methods used in our study. Thirdly, although our NGS panel included a relatively large number of 398 genes associated with growth disorders, causative variants in the genes not present in the panel could have been missed. Last, the subtle syndromic features typical for some genetic diagnoses might be difficult to detect, and the evaluation might be biased by subjective evaluation. Objective methods such as facial recognition software were not available in our study.

## Conclusions

Monogenic etiology is frequent in children with FSS treated with GH, and gene variants affecting the growth plate are the most common. A shorter parent height and the absence of BA delay are the best clinical predictors of monogenic FSS.

## Supplementary Materials

Supplementary table 1

Supplementary table 2 - A table evaluating clinical characteristic and the results of genetic examination in children with proven monogenic etiology of their familial short stature. BA – bone age, CA – calendar age, F – female, GH – growth hormone, LP – likely pathogenic, M – male, (m) – moderate strength of the criterion used, M/- - hemizygote, M/M – homozygote, M/n – heterozygote, NA – not available, NGS – next-generation sequencing, P – pathogenic, SD – standard deviation, (sp) – supporting strength of the criterion used, (st) – strong strength of the criterion used, SHH – sitting height to height, (vs) – very strong strength of the criterion used, WES – whole exome sequencing

Supplementary table 3 – Genetic variants without proven causality

## Declaration of interest

The authors have no conflicts of interest to declare.

## Funding

This work was supported by the Ministry of Health, Czech Republic, grant number NU22J-07-00014.
