# Supplementary material for: Analysis of children with familial short stature: who should be indicated for genetic testing?
Source: Endocr Connect. 2023 Sep 19;12(10):e230238. doi: 10.1530/EC-23-0238 (PMC10563636; doi:10.1530/EC-23-0238)
Supplement: Supplementary table 2 - A table evaluating clinical characteristic and the results of genetic examination in children with proven monogenic etiology of their familial short stature. BA – bone age, CA – calendar age, F – female, GH – growth hormone, LP – likely pathogenic, M – male, (m) – moderate st [file supplementary_table_2.pdf]

Patient	Sex	Age at last follow-up (years)	GH treatment initiation age (years)	GH dose in the first year of GH treatment (ug/kg/day)	Shorter parent's height (SD)	Birth weight (SD)	Birth length (SD)	Height SDS prior to GH treatment	Height SDS after 1 year of GH treatment	Height SDS after 3 years of GH treatment	Growth velocity prior to GH treatment (cm/year)	Growth velocity in the first year of GH treatment (cm/year)	BA prior to GH treatment (difference to CA. years)	IGF1 prior to GH treatment (SD)	Stimulated GH maximum (ug/l)	SHH ratio (SD)	Additional phe-typic features	Genetic examination method	Gene	Mutation status	Transcript variant	Protein variant	Classification	ACMG criteria
**Extracelullar matrix**																								
1	M	14	7	30	-3.6	-1.6	-3.3	-3.7	-2.9	-2.2	3.7	8.6	1.7	-1.6	3.2	0.9	-	Sanger	ACAN	M/n	c.[1425_1425delA. 1425=]	p.Val478fs*14	P	PVS1(vs), PM2(m), PP1(st), PP5(sp)
2	F	9	6	34	-2.9	-1.2	-2.3	-3.3	-2.8	-2.5	2.0	7.6	2.0	-0.1	NA	0.5	-	Sanger	ACAN	M/n	c.916A>T	p.Ser306Cys	P	PM1(m), PM2(m), PP1(st), PP3(sp), PP5(sp)
3	F	13	3	40	-2.4	-1.5	-1.9	-2.8	-2.6	-1.7	7.4	7.3	0.3	-1.3	9.7	2.6	Scoliosis	NGS panel	COL11A1	M/n	c.475A>G	p.Ile159Val	LP	PM1(m), PM2(m), PP1(st)
4	M	16	10	35	-2.0	-1.0	-0.7	-3.4	-2.7	-2.1	3.8	7.8	-1.3	-1.2	8.0	0.7	-	NGS panel	COL11A2	M/n	c.3706C>T	p.Arg1236Cys	LP	PM2(m), PP1(st), PP3(sp)
5	M	18	13	33	-2.8	-2.4	-2.6	-2.7	-2.3	-2.9	5.1	9.0	0.0	0.2	19.6	1.5	Scoliosis	WES	COL11A1	M/n	c.1543C>G	p.Gln515Glu	LP	PM1(m), PM2(m), PP1(m), PP3(sp)

6	M	12	5	35	-2.0	-2.5	-1.5	-3.2	-2.3	-1.9	3.8	9.9	-2.0	-0.9	6.4	-0.2	Frequent long bone fractures, vertabrae compressive fractures	NGS panel	COL1A2	M/n	c.577G>A	p.Gly193Ser	P	PM1(m), PM2(m), PM6(m), PP2(sp), PP3(sp), PP4(sp), PP5(vs)
7	M	16	12	33	-2.9	-2.4	-3.0	-3.4	-2.9	-2.2	4.1	8.7	-1.5	-1.6	8.4	1.2	-	WES	COL2A1	M/n	c.3016C>G	p.Arg1036Gly	LP	PM1(m), PP1(m), PP2(sp), PP3(sp)
8	F	10	8	32	-4.0	-4.0	-2.3	-3.1	-2.6	-2.1	7.8	8.5	-1.8	0.4	0.0	-0.2	-	NGS panel	COL2A1	M/n	c.3106C>G	p.Arg1036Gly	LP	PM1(m), PP1(m), PP2(sp), PP3(sp)
9	F	13	6	32	-2.8	-2.7	-4.1	-3.0	-2.0	-0.8	4.9	10.3	-0.3	-1.0	NA	2.0	Genua valga	WES	COL2A1	M1/ M2	c.410G>A/c.3 106C>G	p.Arg137His/p. Arg1036Gly	LP/ LP	PM1(m), PP1(sp), PP2(s), PP3(sp), PP5(sp)/PM1(m), PP1(m), PP2(sp), PP3(sp)
10	M	5	4	36	-3.3	-1.4	-3.5	-3.1	-2.7	NA	6.8	8.7	-0.3	-1.3	NA	-0.4	Heart murmor	NGS panel	COL2A1	M/n	c.1300C>T	p.Pro434Ser	LP	PM1(m), PP1(st), PP2(sp), PP3(sp)
11	M	8	4	33	-3.2	-1.9	-2.1	-3.6	-3.5	NA	4.9	6.0	1.4	-2.2	17.1	0.3	Limited elbow extension, pes planum, saber-like forarm and tibia	NGS panel	COL2A1	M/n	c.2129C>T	p.Pro710Leu	LP	PM1(m), PM2(m), PP1(m), PP2(sp), PP3(sp)
12	M	16	NA	NA	-4.2	-2.6	NA	-3.3	NA	NA	NA	NA	NA	-1.7	NA	7.6	Genua vara	NGS panel	COMP	M/n	c.1220G>A	p.Cys407Tyr	LP	PM1(m), PM2(m), PP1(m), PM5(m), PP2(sp), PP3(sp)
13	M	10	9	31	-2.3	-2.2	-2.4	-2.4	-1.6	NA	5.5	9.2	0.1	0.1	NA	1.0	Little finger cli-dactyly	NGS panel	MATN3	M/n	c.671G>A	p.Arg224Gln	LP	PM1(m), PM2(sp), PP1(m), PP3(sp)
**Paracrinne growth plate regulation**																								
14	M	9	5	36	-2.9	-0.6	-1.0	-4.4	-4.1	-3.9	7.6	7.2	-1.6	-1.7	9.4	3.6	-	WES	FGFR3	M/n	c.1612A>G	p.Ile538Val	P	PM1(m), PM2(m), PP2(sp), PP3(sp), PP5 (st)
15	F	5	4	35	-2.4	-1.6	-2.4	-3.0	-2.9	NA	3.7	6.7	NA	-2.1	4.0	0.3	-	NGS panel	FGFR3	M/n	c.251C>T	p.Ser84Leu	LP	PM2(m), PP1(sp), PP2(sp), PP5(sp)
16	M	9	7	32	-2.4	0.9	-1.7	-2.9	-2.2	NA	5.6	10.3	-1.7	-1.4	4.7	0.3	-	NGS panel	NPR2	M/n	c.613C>T	p.Arg205*	P	PVS1(vs), PM2(m), PP3(sp)
17	F	15	10	36	-2.0	-1.4	-1.2	-3.0	-2.7	NA	4.9	7.3	-2.1	-1.6	5.8	2.2	Brachydactyly	NGS panel	NPR2	M/n	c.1673T>C	p.Ile558Thr	P	PM1(m), PM2(m), PP1(st), PP2(sp), PP3(sp)
18	M	10	5	32	-3.7	-2.0	-3.1	-4.0	-3.3	-2.5	4.3	8.4	-1.1	-0.7	4.5	1.2	Foramen ovale apertum	NGS panel	NPR2	M/n	c.1808G>C	p.Ser603Thr	LP	PM1(sp), PM2(st), PP2(sp), PP3(sp)

19	F	11	3	37	-2.4	-1.8	-2.0	-3.8	-3.1	-2.6	5.4	9.0	NA	-1.7	12.7	1.0	Pes planum	NGS panel	NPR2	M/n	c.1670G>A	p.Arg557His	LP	PM1(m), PM2(sp), PP1(m), PP2(sp), PP3(sp)
**Fundamental Intracellular processes of the growth plate**																							
20	M	12	8	33	-2.9	-2.4	-3.4	-2.7	-2.0	-1.3	4.6	9.0	0.5	-1.0	5.9	0.5	-	WES	EXT2	M/n	c.2034G>C	p.Lys678Asn	P	PVS1(vs), PM2(st), PP1(m), PP2(sp), PP3(sp)
21	M	16	11	33	-2.9	1.0	-1.2	-3.9	-2.9	-2.6	2.5	9.2	0.5	-4.3	6.2	0.7	Vitiligo	Sanger	PTPN11	M/n	c.211T>A	p.Phe71Ile	P	PM1(st), PM2(m), PM5(st), PP2(sp), PP3(sp)
22	M	12	7	33				-2.7	-1.9	-1.2	4.0	9.3	-1.6	-1.4	4.8	1.5	-	Sanger	PTPN11	M/n	c.1403C>T	p.Thr468Met	P	PS3(vs), PM1(m), PM2(m), PM5(st), PP2(sp), PP3(sp), PP5(m))
23	F	12	6	33	-3.9	1.0	0.1	-2.5	-1.7	-1.0	4.8	8.7	-0.7	-1.1	1.2	?	Café-au-lait spots, hamartomas in brain	Sanger	NF1	M/n	c.4267A>G	p.Lys1423Glu	P	PS3(st), PM1(m), PM2(st), PM5(st), PP2(sp), PP3(st), PP5(st)
24	M	13	7	46	-3.4	-1.6	-1.9	-2.1	-1.3	-0.4	6.0	9.6	-1.1	-0.5	NA	2.2	Saber like forearm, gothic palate	MLPA	SHOX	M/n	SHOX deletion (1MB)	-	P	X
25	M	13	6	33	-2.4	-1.6	-1.7	-2.7	-2.2	-1.8	5.3	8.0	-0.2	-2.1	NA	-0.6	-	MLPA	SHOX	M/n	46.X inv dup (Y) (pterp 11.1)	-	P	X
26	M	17	11	52	-3.2	-1.0	-2.4	-2.9	-2.3	-2.1	3.4	9.5	1.5	-0.6	NA	0.4	-	MLPA	SHOX	M/n	PAR1 duplication	-	P	X
27	F	4	1	48	-2.9	-0.2	-3.5	-2.5	-2.3	NA	8.9	9.7	NA	-1.4	NA	2.4	-	FISH	SHOX	M/n	del Xp22.3	-	P	X
28	F	15	12	49	-2.9	-1.1	-1.0	-2.7	-2.3	-1.8	7.8	8.9	-0.3	-1.6	NA	4.1	-	FISH	SHOX	M/n	del Xp22.3	-	P	X
29	M	9	6	48	-3.5	-0.5	-1.1	-2.6	-1.8	-0.9	5.1	10.6	NA	-1.1	NA	?	-	MLPA	SHOX	M/n	del Xp22.3	-	P	X
[redacted]																								
30	F	10	7	31	-2.8	-2.6	-3.1	-3.2	-2.1	-1.4	4.2	10.6	-0.6	-2.1	7.2	0.1	-	WES	GHSR	M/n	c.526G>A	p.Gly176Arg	LP	PM1(sp), PM2(m), PP1(sp), PP3(sp)
31	M	7	4	33	-3.9	-2.0	-1.4	-3.1	-2.4	-1.9	5.1	9.1	-2.0	-2.2	8.3	0.7	Strabism	WES	HMGA2	M/n	c.223C>T	p.Arg75Trp	LP	PM1(m), PM2(m), PP1(m), PP3(sp)

32	M	11	7	34	-2.8	-1.6	-1.7	-3.5	-2.6	-1.6	5.3	9.8	-0.2	-1.8	6.1	0.6	-	WES	IGFALS	M/n	c.589C>T	p.Arg197Cys	LP	PP1(st), PP2(sp), PM2(sp)
33	M	10	6	28	-2.8	-1.2	-1.1	-6.3	-5.0	-3.4	4.5	12.1	0.0	<-3	1.7	0.4	Psychomotoric retardation	NGS panel	OTX2	M/n	c.106delC	p.Arg36fs	P	PVS1(vs), PM2(m), PP3(sp), PP5(sp)
[redacted]																								
34	M	10	5	34	-2.7	-1.1	-1.8	-4.4	-4.0	-3.6	5.9	7.2	NA	-1.1	6.4	0.9	Immu-deficiency, Hyperkeratosis, cornea scarring	WES	MBTPS2	M/-	c.1538T>C	p.Leu513Pro	LP	PM1(m), PM2(m), PP2(sp), PP3(sp), PP5(sp)
35	M	12	7	34	-2.4	-1.8	-1.4	-2.8	-2.1	-1.0	6.3	8.7	NA	-1.0	4.6	-0.3	Radial ray defect. right kidney dystopia	WES	SALL4	M/n	c.1717C>T	p.Arg573*	P	PVS1(vs), PM2(m), PP1(sp), PP3(sp), PP4(sp)
36	M	16	12	33	-2.9	-1.6	-2.4	-4.0	-3.6	-3.3	4.2	7.6	-3.1	-3.7	10.0	2.6	Central hypothyroidism	WES	TRHR	M/M	c.392T>C	p.Ile131Thr	P	PM1(m), PM2(m), PS3(m), PP3(sp), PP4(sp)

Supplementary table 2 - A table evaluating clinical characteristic and the results of genetic examination in children with proven monogenic etiology of their familial short stature. BA – bone age, CA – calendar age, F – female, GH – growth hormone, LP – likely pathogenic, M – male, (m) – moderate strength of the criterion used, M/- - hemizygote, M/M – homozygote, M/n – heterozygote, NA – not available, NGS – next-generation sequencing, P – pathogenic, SD – standard deviation, (sp) – supporting strength of the criterion used, (st) – strong strength of the criterion used, SHH – sitting height to height, (vs) – very strong strength of the criterion used, WES – whole exome sequencing
