# Supplementary material for: Analysis of children with familial short stature: who should be indicated for genetic testing?
Source: Endocr Connect. 2023 Sep 19;12(10):e230238. doi: 10.1530/EC-23-0238 (PMC10563636; doi:10.1530/EC-23-0238)
Supplement: Supplementary table 3 – Genetic variants without proven causality [file supplementary_table_3.pdf]

Supplementary table 3 – Genetic variants without proven causality

Gene	Transcript variant	Protein variant	Mutation status	ACMG classification
ALPL	c.40C>T	p.Leu14Phe	M/n	Benign
COL10A1	c.1518G>C	p.Lys527Asn	M/n	Likely benign
COL10A1	c.773G>A	p.Arg258Gln	M/n	Uncertain significance
COL11A1	c.1896G>A	p.Met632Ile	M/n	Uncertain significance
COL1A2	c.2642A>C	p.Glu881Ala	M/n	Uncertain significance
COL1A2	c.2798A>G	p.Asp933Gly	M/n	Benign
COL1A2	c.2777G>A	p.Arg926His	M/n	Uncertain significance
COL2A1	c.3713A>C	p.Tyr1238Ser	M/n	Likely benign
COL9A2	c.1834G>A	p.Gly612Arg	M/n	Benign
COL9A2	c.1798C>T	p.Arg600Cys	M/n	Uncertain significance
COL9A2	c.185C>T	p.Pro62Leu	M/n	Uncertain significance
COL9A2	c.1693G>A	p.Gly565Arg	M/n	Benign
COMP	c.1480G>C	p.Asp494His	M/n	Benign
EXT1	c.947G>A	p.Arg325Gln	M/n	Uncertain significance
FBLN1	c.1174G>A	p.Gly392Ser	M/n	Uncertain significance
FBN1	c.902G>T	p.Gly301Val	M/n	Uncertain significance
FGFR1	c.2292+3A>G	intronic	M/n	Likely benign
FLNA	c.1463G>A	p.Arg488Gln	M/-	Uncertain significance
FLNB	c.731C>T	p.Pro244Leu	M/n	Uncertain significance
FLNB	c.2935G>A	p.Val979Met	M/n	Likely benign
GH1	c.478C>T	p.Arg160Trp	M/n	Benign
GH1	c.171+2T>A	Splice site	M/n	Uncertain significance
GHSR	c.709A>T	p.Arg237Trp	M/n	Uncertain significance
GLI2	c.4332G>A	p.Met1444Ile	M/n	Benign
GLI3	c.1222G>A	p.Gly408Ser	M/n	Uncertain significance
HSPG2	c.12874G>A	p.Glu4292Lys	M/n	Benign
IGF1R	c.394A>C	p.Asn132His	M/n	Benign
IGFALS	c.589C>T	p.Arg197Cys	M/n	Uncertain significance

IGFALS	c.860C>T	p.Pro287Leu	M/n	Uncertain significance
IHH	c.1169G>A	p.Arg390His	M/n	Benign
IHH	c.857C>T	p.Pro286Leu	M/n	Uncertain significance
IHH	c.1169G>A	p.Arg390His	M/n	Benign
LTBPS3	c.2222C>G	p.Ala741Gly	M/n	Likely benign
PAX6	c.13C>G	p.His5Asp	M/n	Benign
SHH	c.424G>A	p.Glu142Lys	M/n	Benign
SOST	c.448G>C	p.Gly150Arg	M/n	Likely benign
SOX9	c.817G>C	p.Val273Leu	M/n	Likely benign
THRA	c.455G>A	p.Arg152Gln	M/n	Uncertain significance
TRPS1	c.793A>G	p.Asn265Asp	M/n	Uncertain significance

M/n heterozygote, M/- hemizogote
